# I am in the passenger seat of my own body: a qualitative interview study of the relationship between binge eating and concurrent problematic alcohol use

**DOI:** 10.1186/s40337-026-01576-z

**Published:** 2026-03-16

**Authors:** Magdalena Jansson, Lovisa Olsson, Anne-Charlotte Wiberg, Thomas Parling, Ata Ghaderi, Stina Ingesson-Hammarberg

**Affiliations:** 1 Centre for Psychiatry Research, Department of Clinical Neuroscience, Karolinska Institutet, & Stockholm Health Care Services, Region Stockholm, Norra Stationsgatan 69, Stockholm, 113 64 Sweden; 2 Department of Clinical Neuroscience, Karolinska Institutet, Stockholm, Sweden

**Keywords:** Binge eating, Problematic alcohol use, Emotion regulation difficulties, Comorbidity, Integrated treatment

## Abstract

**Background:**

The association between binge eating and alcohol use disorders (AUDs) has been demonstrated in genetic, population-based, and clinical studies. Individuals with binge eating report more binge drinking and heavier alcohol consumption than those with eating disorders (EDs) without binge eating. Co-occurring EDs and AUDs are linked to higher levels of ED symptoms, depression, anxiety, and more severe psychosocial impairment compared with EDs alone. The aim of this qualitative study was to explore how adults with concurrent binge eating and perceived alcohol problems experience these two conditions and their potential interrelationship. Such insight is needed to improve understanding of functional links and perpetuating processes that may complicate treatment.

**Methods:**

Twelve adults with concurrent binge eating and problematic alcohol use were recruited from a public specialized ED clinic in Stockholm, Sweden, or through an ED patient-organization website between 2024 and early 2025. All interviews were analyzed using reflexive thematic analysis following the principle of Braun and Clarke.

**Results:**

Four themes were identified. Binge eating and problematic alcohol use were shown to be closely interconnected, as co-occurring emotion regulation strategies, and alcohol being involved before and as part of binge eating situations. Alcohol use also contributed to maintaining the eating disorder, for example, by providing temporary relief from self-critical thoughts and strict food rules, or by serving as a coping mechanism to reduce body shame in social situations. Negative responses from healthcare providers regarding concurrent alcohol problems hindered appropriate treatment seeking for both conditions.

**Conclusions:**

These findings demonstrate the functional relationship between binge eating, and alcohol use, and the maintenance processes of the two conditions. Integrated treatment for both conditions, as well as preventive efforts through earlier identification of alcohol problems in individuals with binge eating difficulties are warranted.

**Supplementary Information:**

The online version contains supplementary material available at 10.1186/s40337-026-01576-z.

## Abstract (plain language summary)

Studies in both the general population and in patients seeking treatment for eating disorders show that binge eating and alcohol problems often occur together. When they co-occur, people tend to experience more severe eating-disorder symptoms, and treatment can become more challenging. This study explored how adults who struggle with both binge eating and problematic alcohol use understand these difficulties and how they influence one another. The goal was to deepen understanding of how the two conditions may be interconnected and to generate ideas for improving current eating-disorder treatments based on these insights.

Twelve adults were interviewed about their experiences with having both binge eating and alcohol problems. They were recruited either at a specialized eating-disorder clinic or through a patient-organization website for eating disorders. The interviews were analyzed to identify themes that represented participants’ views of the how they experienced the relationship between binge eating and alcohol problems.

Participants described binge eating and alcohol use as closely connected. Both were used to cope with difficult emotions, and alcohol often played a role before or during binge-eating episodes. Alcohol use also contributed to maintaining the eating disorder, for example, by providing temporary relief from self-critical thoughts and strict food rules, or as a coping mechanism in reducing body shame in social situations. Participants also reported that negative reactions from healthcare providers to their alcohol use made it harder to seek or receive appropriate help for either problem.

These findings demonstrate the need for treatment approaches that address binge eating and alcohol use together, and potential shared underlying aspects that contribute to maintaining both disorders. Such interventions are needed to increase outcomes, and retention in treatment as well as earlier detection of alcohol-related difficulties in people with binge eating problems.

**Supplementary Information:**

The online version contains supplementary material available at 10.1186/s40337-026-01576-z.

## Background

In Western countries, the lifetime prevalence of eating disorders (EDs) among women is approximately 8–18%, with higher rates observed in adolescent and young adults [1, 2]. Psychiatric comorbidities are highly common in EDs, with up to 70% meeting the criteria for a concurrent diagnosis such as anxiety, depression, neuropsychiatric disorders, or substance use disorders (SUDs) [3, 4]. Among these, alcohol use disorders (AUDs) are particularly prevalent. The association between binge eating and alcohol use disorders has been identified in genetic, population, and clinical studies [3, 5–9]. Individuals with binge eating report more binge drinking, and heavier alcohol consumption patterns, as compared to individuals with EDs that do not include binge eating. This pattern includes individuals with anorexia nervosa with binge eating and purging, who have a higher frequency of problematic alcohol use, as compared to those with AN without binge eating/purging [10]. Individuals with both EDs and AUDs exhibit higher rates of ED symptoms, depression, and anxiety, as well as more severe psychosocial difficulties and poorer social functioning than those with an ED alone [5, 11]. Hence, it is particularly important to better understand the clinical implications of treating patients with comorbid EDs and comorbid conditions, in general, and problematic alcohol use in particular.

Emotion regulation difficulties are one shared feature that has been explored to understand the more complexconditions including both EDs and alcohol problems. Such investigations have shown that both binge eating and problem drinking are maintained by their function through negative reinforcement, which is often about relieving negative affects [12–15], such as anxiety, anhedonia, or guilt prior to binge eating, and/or drinking [14]. This may be driven by specific difficulties in regulating emotions, as well as impulsivity which are shared features in EDs and AUDs [6, 14, 16–18]. More specifically, difficulty in being able to identify, being confused, or overwhelmed by feelings, and the tendency to suppress and avoid feelings, have been positively associated with both EDs and SUDs [15, 19–21].

Another plausible explanation for the high co-occurrence of binge eating and AUDs, is the limited treatment access and long delays before receiving care for both EDs and AUDs. Reported barriers include failure to recognize the perceived symptoms as mental health problems, fear of stigma, and limited treatment availability and options [22–26]. Such barriers may prolong the time with the disorder, contributing to both more severe ED and AUD symptoms and the development of secondary conditions in both directions. Another aspect that may explain the relationship between binge eating and AUDs is that other psychiatric disorders may contribute to the development of comorbid conditions. One proposed link between binge eating and AUDs is the presence of either post-traumatic stress disorder and/or major depressive disorder, as mediators of the relationship between binge eating behaviors and AUDs [9, 27].

Individual perspectives on the mechanisms behind how these problems are connected and maintained are lacking, and are a current gap in research. Using qualitative methods in the current study has the potential to provide in-depth information on the thoughts, feelings, and individual subjective experiences of having both binge eating and alcohol problems. Qualitative interviews, as compared to quantitative data, can provide information on how the participants interpret these experiences, but also further understand the contexts and life situations in which the participants have these experiences. To our knowledge, there is no qualitative interview study that has explored how individuals who simultaneously suffer from EDs (including binge-eating) and alcohol problems perceive these conditions, their potential interconnectedness, and their consequences. The aim of this qualitative study was to explore how adults with concurrent binge eating and perceived alcohol problems, experience these two conditions, and their potential relationship (e.g., whether they potentially maintain each other) from an individual perspective. Such an investigation is warranted for improving our understanding of potential functional relationships and perpetuating processes in binge eating and alcohol use that make them more complex to treat. It may also generate further hypotheses for developing a novel integrated treatment approach for binge eating and alcohol problems.

## Methods

### Design

This was a qualitative interview study including 12 adults who had a concurrent problem with binge eating, and problematic alcohol use. The interviews were conducted in 2024–2025, in Stockholm, Sweden. The current study was approved by the Swedish Ethical Review Authority (No. 2024-05329-01; 2024-07895-02). The manuscript is reported in line with Consolidated Criteria for Reporting Qualitative Research (COREQ) [28].

### Participants and procedure

Participants were recruited as a purposive sample at the Stockholm Centre for Eating Disorders, a public clinic specializing in ED treatment, through advertisements in waiting rooms and by handouts from therapists. Advertisements were also posted on the social media account of SHEDO, a Swedish patient organization for individuals with EDs and/or deliberate self-harm. Eligible participants were individuals above 18 years of age, who identified as having both binge eating and concurrent alcohol problems, with no diagnostic procedure included. Exclusion criterion was insufficient proficiency in Swedish. Those who reported an interest in participation were contacted by email, or phone, to schedule an interview appointment. Interviews were conducted either at the Stockholm Centre for Eating disorders, or by video meeting, using an end-to-end encrypted video conferencing platform. In the scheduled meeting, eligible participants received written and oral information about the study and were assessed for eligibility by one of the researchers. After being included, participants reported on their alcohol consumption the previous 30 days, together with the researcher, in line with the timeline follow-back method (TLFB) [29]. The last and the second author performed the interviews, after testing the interview guide, with no changes, after the first interview. All interviews were recorded and transcribed verbatim. No additional notes were taken or included in the data collection, and no follow-up interviews were scheduled. No transcripts were returned to the participants. The participants who came to Stockholm Centre for Eating Disorders (*n* = 2) filled out questionnaires after finalizing the interview. Those who participated via video meeting (*n* = 10), received the questionnaires by mail after the interview, filled them out at home, and sent them back to the researcher. No participant withdrew their consent during or after the interview. All participants received two cinema tickets as reimbursement for their participation.

### Measures

Participants reported on the following self-report measures: The Eating Disorder Examination Questionnaire [30] was used to measure ED symptom severity. The Clinical Impairment Assessment [31] was used to assess the level of psychosocial impairment due to the ED [32, 33]. The degree of alcohol problems was measured with the Alcohol Use Disorders Identification Test (AUDIT) [34]. Emotion regulation difficulties were assessed with the brief Difficulties in Emotion Regulation Scale (DERS-16) [35]. Depressive and anxiety symptoms were assessed with the Patient Health Questionnaire (PHQ) [36], and Generalized Anxiety Disorder Scale (GAD-7) [37]. Alcohol consumption was measured with the TLFB [29], including the previous 30 days. Lastly, participants reported on sociodemographic data, e.g. occupational- and relationship status.

### Interview guide

The interview was semi-structured and included three main questions and allowed for follow-up questions from the researcher. The questions covered; (1) how participants perceived their binge eating and alcohol problem; (2) if they seemed to be connected in some way, and if so, how, and (3) if either the binge eating or alcohol use, maintained the other problem. The researcher encouraged the participants to speak freely and express their opinions on the different topics (Please see supplementary for the complete interview guide).

### Researchers’ stance

The research team brought diverse but complementary clinical and methodological backgrounds to the study. All authors were experienced clinicians from the alcohol, and eating disorder field, which shaped the formulation of the research questions, the conduct of the interviews, and the interpretation of the data. This clinical familiarity facilitated sensitivity to participants’ narratives but also required ongoing reflexive awareness to avoid premature interpretations based on prior professional assumptions. Reflexivity was actively addressed throughout the analytic process by discussing alternative interpretations within the research team. The involvement of researchers at different career stages further supported critical reflection and methodological rigor in the thematic analysis.

### Analytical framework

The chosen method for the analysis was reflexive thematic analysis according to the principles outlined by Braun and Clarke [32, 38], in which themes relevant to the research questions were identified through a six-step process. The analytical procedure was as follows, each author independently read the interview transcripts to become familiar with the material. The first, second, and last author separately identified codes, i.e. specific words, sentences or sections which were of interest to the research questions. Both explicit and latent codes were used. A preliminary codebook was created by the first and second author independently, and these were reviewed, discussed, and revised in collaboration with the last author. Together, they organized the codes into themes. These themes were then reexamined and refined through ongoing discussions with the rest of the research team. This process continued until the themes were clearly defined. In the final stages, SIH took primary responsibility for drafting the manuscript, with input and feedback from the other authors.

### Statistical analyses

Statistical analyses included basic descriptive statistics to describe the study participants in terms of background variables and clinical characteristics. The participants’ weekly alcohol consumption was calculated as the total number of weekly standard drinks in the 30-day period, divided by the number of weeks (4.29). Analyses were performed using SPSS version 31 [39].

### Sample size estimation

The interview study planned for 30 participants as the maximum but had no further estimation of the expected number of interviews beforehand. When reaching 12 participants, we deemed the information power in the material to be high, i.e. that the data material contained rich, in-depth information with the ability to answer the research questions [40].

## Results

The study included 12 participants, 10 women, one man, and one person who identified as non-binary, and their mean age was 39.7 years. The interviews lasted 23–70 min (mean 43.5 min). All participants had sought treatment for the ED, and two participants had experience from addiction treatment (not displayed in table). Three participants were on sick leave, and the rest were either students or employed (See Table 1).

**Table 1:** Sociodemographic and clinical characteristics of the interview sample (*n* = 12)

Gender (% female)	83.3%		
	M	SD	Range
Age	39.67	13.01	22–60
	N (%)		
Educational status			
Up to secondary school	3 (25)		
Post-secondary school	2 (16.7)		
University studies < 3 years	3 (25)		
University > 3 years	4 (33.3)		
Employment status	N (%)		
Employed/self-employed	5 (41.7)		
Sick leave	4 (33.3)		
Unemployed	1 (8.3)		
Student	2 (16.7)		
Relationship status	N (%)		
Single	7 (58.3)		
Partner/married	5 (41.7)		
EDE-Q	3.97	0.9	2.16–5.07
CIA	34.42	9.77	12–47
Weekly number of drinks (TLFB 30)	7.13	7.61	0-19.63
AUDIT	12.58	7.01	0–22
GAD-7	14.00	6.38	5–21
PHQ-9	16.17	4.88	7–22
DERS-16	61.17	12.86	36–76

EDE-Q = Eating Disorder Examination Questionnaire; CIA = Clinical Impairment Assessment; TLFB = Timeline follow back 30 days; AUDIT = Alcohol Use Disorder Identification Test; GAD-7 = Generalized Anxiety Disorder Assessment; PHQ-9 = Patient Health Questionnaire; DERS-16 = Difficulties in Emotion Regulation Scale - Brief version

The analysis resulted in four identified themes. For a schematic overview of the themes see Table 2.

**Table 2:** Schematic overview of themes, examples of codes and quotes of the respective themes

Theme	Code (example)	Quote (example)
Binge eating and alcohol are alternating ways to handle strong emotions	Escape from painful feelings Get into the bubble Numb myself	*Well*,* I’ve realised*,* for me*,* it’s an escape. /… / It has been my refuge when I can’t stand it*,* can’t take it anymore. I just want to get into my bubble; I just have to cut myself off from everything and everyone.”*
Taking a break from control	Dared to let go of control when intoxicated Could enjoy eating when being drunk	*“One can let go of the eating disorder*,* when drunk. Well*,* it was less of a priority*,* once being drunk to a certain level*,* I could let it go. And when I was drunk enough*,* I could just eat and enjoy it.”*
From binge eating to drinking alcohol – managing to keep on with the ED	Alcohol was used to handle feelings of weight-related shame	*“I didn’t have a problem with alcohol until I started binge eating again. /… / And I guess that’s my way of getting my feelings out and getting some comfort. It was so hard to deal with*,* because I saw the change in my body. So*,* then I started drinking. To try to escape it.”*
Problem drinking as a barrier to appropriate treatment	Participant felt misunderstood when remitted to dependency care	*“They thought that I should get help with it (the alcohol) first. And then I was just disappointed and sad and then I ditched it all. Because it’s the same problem*,* I think. But it’s the binge eating that is my foundation*,* my comfort blanket that I’ve had for so very long. It felt like the completely wrong end to start”*

### Binge eating and alcohol are alternating ways to handle strong emotions

All participants mentioned binge eating or drinking alcohol as ways to handle difficult and negative emotions. Participants typically described processes of emotional build-up of negative feelings, such as anxiety, sadness, depressive moods, anger, shame, guilt, or pain. They described similar processes of emotions building up stronger and stronger, and that eating and drinking made it possible to escape from these feelings. There were also several examples of binge eating and drinking similarly serving as ways to handle both acute emotionally triggering events, such as interpersonal conflicts, or more general long-term strain due to stressful life situations, or traumatic life events. Several participants described the start of binge eating as a process of giving in to either, the urge, or strong emotion, and stepping into a bubble, or a safe space, where the preceding emotions subside, and nothing else is present, or matters. Just before the start of binge eating, when reaching the decision to binge eat, participants experienced a relief of negative emotions, or being calm. Further, when engaging in eating, participants described eating as relaxing, all feelings from before disappearing, and being in a private safe space, or room, where no demands or expectations from the outer world are present. Being in the bubble involved drinking for many of the participants, although both behaviors were not always mixed.

*“Well*,* I’ve realised*,* for me*,* it’s an escape. /… / It has been my refuge when I can’t stand it*,* can’t take it anymore. I just want to get into my bubble; I just have to cut myself off from everything and everyone.”* (Participant 10).

*” I don’t have to think and feel*,* I can sit and eat and watch TV and then I push everything else aside. And then I don’t have to have the feeling of loneliness or that I have needs that I think I’m not allowed to have. That helps me push things aside.”* (Participant 4).

Emotional buildup that resulted in binge eating and/or drinking could also be characterized as physical experiences, such as an itch, or physical tension. Such physical tension were experienced, sometimes following periods of stricter food restriction.

*“I get restless. Extremely restless. It’s like my body itches. Often*,* I’ve been annoyed with someone at work /… / or it has been several days without me binge eating. It’s like it’s building up. It’s like it’s stored somehow*,* and then I must let go. Or*,* that it has to ooze out somehow. /… / all the negative feelings. Stress and irritation*,* and it doesn’t go away if I don’t binge eat or drink.”* (Participant 2).

Some participants described binge eating and alcohol as alternating problem behaviors, depending on the situation, or as something they noticed were changing over time. For example, trying to change one behavior, resulted in an increase of the other. One participant noticed changes when e.g. initiating treatment for the ED. When focusing specifically on regular eating, this initially increased drinking alcohol, as the eating was no longer a way of releasing emotional tensions. The process could also be over longer periods of time, where others experienced periods in life to be more dominated by the ED, and in other times, problematic alcohol use, but that both binge eating and drinking still served the purpose of suppressing intolerable emotions. One participant described more frequent changes in problem behaviors, with a typical pattern with alternating behaviors, depending on her family situation. Alcohol could be involved or a distinct problem behavior, but in most participants, both alcohol and binge eating were involved in the same problem situations.

*“They went very hand in hand in some way*,* I did both*,* I ate and drank at the same time*,* ate a lot of sweets and stuff and drank wine at the same time*,* it wasn’t one or the other there*,* but they were together those years*,* I think. It was a double way to numb myself in some way and go into this bubble then /… / when I wanted to just disappear a bit.”* (Participant 10).

In contrast to their role in relieving negative affect, some participants also described binge eating and alcohol use as comforting and rewarding, and sometimes actively anticipated behaviors. Alcohol was more often associated with social occasions, rewarding oneself after hard work, or a stressful work situation, and sometimes even as a reward for maintaining food restriction. Likewise, several participants described the anticipation and ritual of binge eating, including planning, purchasing or preparing food, as something pleasurable that they could look forward to. For some, the positive emotions were intensified by the intention to purge afterward. Planning to purge afterwards created a sense of not having to worry about consequences but also allowing them to feel less conflicted about indulging in both food and alcohol. One participant explained that it was not always negative emotions leading up to binge eating; sometimes eating was associated with feelings of happiness, wanting to get a thrill, having a good time and celebrating herself and reinforcing those feelings. Another common description was the use of food and alcohol to create space for oneself. These moments were described in the sence that initially, eating and drinking served comforting or rewarding purposes. After a while, this could change, and become an episode of binge eating.

### Taking a break from control

Participants described that alcohol and the EDs were connected in the way that drinking was a way to cope with the mental strain of the EDs. Constant worrying about eating, planning, as well as comparing oneself to others, negative thoughts related to appearance, or self-doubt were triggers to drinking as alcohol provided a temporary escape from these thoughts. Drinking provided a temporary break from the “control regimen”, but it could also result in letting go and eating more than expected. Alcohol was used both as something that preceded or followed upon binge eating.

Several participants described their view of themselves as harsh, critical, or punishing, and that drinking let them both enjoy eating, without being self-critical, or being able to eat without compensating afterwards.

*“One can let go of the eating disorder*,* when drunk. Well*,* it was less of a priority*,* once being drunk to a certain level*,* I could let it go. And when I was drunk enough*,* I could just eat and enjoy it.”* (Participant 1).

Alternating between restrictive eating and binge eating, abstaining, and losing control over eating and drinking were perceived as somewhat similar experiences. One common example was eating more than intended, or breaking a specific eating rule, which commonly resulted in loss of control over eating. Further, several participants described that they never had been able to limit alcohol, and that both alcohol and eating resulted in loss of control regarding the other problem.

*“When I do binge eat*,* it doesn’t feel like I’m in control*,* it feels like I’m sitting in the passenger seat of my own body. And it becomes black in front of my eyes*,* it’s like*,* everything is consumed even though in the beginning I thought*,* of course that I don’t want this. Of course*,* I don’t want to eat so much that I feel ill and of course I don’t want those feelings that come after. But once you’re in it*,* it doesn’t stop. Until it’s done. /… / And then with the alcohol it’s a bit similar*,* I usually always think; God*,* I don’t want to drink so much*,* so that I feel ill or that I embarrass myself because it’s just awful. But once I’ve started*,* I think it’s kind of the same thing.”* (Participant 8).

Some participants described mixing binge eating and alcohol intensely, which seemed to amplify the feelings of impaired control, and that binge eating got more intense and longer when drinking. One typical chain of losing control was drinking, which commonly lead to drinking more than intended, and then eating more than intended, which in turn would lead to binge eating. Such episodes were sometimes followed by drinking as the binge eating proceeded and lastly, drinking after purging to regulate emotions of guilt and shame and be able to sleep. Such days were then followed by a new day with increased control and restriction to compensate for the impaired control in both eating and drinking, which in turn would increase the risk of binge eating again.

*It often happened that I felt I had a good day when it came to food. I hadn’t eaten too little or too much. Then I was going out to dinner with my family*,* and maybe I’d have a glass of wine or two*,* just because I was happy and it tasted good. But after a third glass*,* I would sometimes start to feel like I had eaten too much. I don’t really know if that was true every time*,* but the feeling came anyway. And sometimes I did eat more than I had planned*,* clearly so. In those moments*,* it could feel like my “good day” was ruined*,* and that I needed to compensate for it afterwards.* (Participant 11).

In other cases, binge eating came first, but led to excessive drinking during, and after, to handle the emotional responses after binge eating.

The ongoing process of maintaining control over eating, and the ED related cognitions, sometimes resulted in intentionally losing control over alcohol. Also, participants described losing control with alcohol as a way of letting go of trying to control stressful life situations or perfectionistic attitudes toward oneself. Some participants reflected on how this surrender of control felt necessary, even if it was unwanted.

*With binge eating*,* it’s about losing control. Or letting go of control. In a way*,* it is a choice. It’s different with alcohol. Then I wanted to let go. And it could be more of a social thing*,* to socialize with friends.* “…” *“I think it tastes like crap*,* but once you feel a sense of well-being. When you get drunk*,* you want it more. Once I’ve gotten into that little bit of drunkenness*,* shit*,* then I want more. And drank before I had eaten anything. Because I wanted the intoxication to come faster as well.*” (Participant 1).

Another aspect described by most participants was the feeling of not being able to stop or ever being able to interrupt a binge or drinking episode. Episodes usually continued until they physically could not eat or drink any more. The most frequent description of how a binge or drinking episode would come to an end was by passing out, falling asleep, or vomiting. Further, the tendency to let go, also had negative consequences of the day after, where participants experienced a stronger tendency to let go of control of eating, due to tiredness or being hungover.

### From binge eating to drinking alcohol – managing to keep on with the eating disorder

All participant described how their EDs preceded their alcohol problems. Many had not had contact with alcohol until they were young adults whilst their ED had emerged in childhood or early teenage years. This relationship between the ED and alcohol use, was explained in different ways. For some, alcohol soon became a way to handle secondary consequences of the ED, including shame about weight gain, and/or guilt caused by eating behaviors, or a general sense of self-loathing, shame, negative feelings about oneself, or loneliness.

*“I didn’t have a problem with alcohol until I started binge eating again. /… / And I guess that’s my way of getting my feelings out and getting some comfort. It was so hard to deal with*,* because I saw the change in my body. So*,* then I started drinking. To try to escape it.”* (Participant 8).

Others described alcohol use as a response to emotional or situational overload, where their ED alone was no longer sufficient to manage all the emotions or life circumstances. Another participant reflected on how alcohol came into the picture after a long-standing reliance on binge eating to handle negative feelings and life situations. One participant described it as a general vulnerability where alcohol and the ED in general were just parallel symptoms of being unable to cope with the underlying problems.

*“If you have an alcohol abuse*,* there is usually something underneath that has started that addiction*,* I think. In my case*,* it is binge eating. That you have to have something all the time. And then I happened to get into alcohol”* (Participant 7).

Participants also noticed that the alcohol problem arose due to their concerns about the impacts of the ED, such as weight gain. For them, drinking was a means to reduce hunger, and body sensations related to hunger, and thereby maintaining food restriction in the short term. Further, alcohol was also used to manage social situations. For instance, they drank alcohol to be able to break isolation, to be able to go out with friends, or meet with a romantic partner, without being completely preoccupied with their appearance.

*“Alcohol helps me do what I want*,* even when I feel the way I do. After being isolated for a couple of days*,* sitting at home and eating*,* something starts smoldering inside me. I start to feel that I want to belong too — to have fun*,* laugh*,* and hang out with people. When I’m sober*,* I feel like I can’t do that because of how I look*,* and then alcohol becomes my solution. It helps me get what I want*,* even though I don’t think I can manage it when I’m sober — at least not completely*,* not the way I’d like to.”* (Participant 4).

### Problem drinking as a barrier to appropriate treatment

Several participants described that they had stopped drinking alcohol after experiencing too many negative consequences, affecting their social and personal life or safety. This contrasted to their experience with the ED, which none of the participants believed they would be able to recover from on their own. Participants had primarily received treatment for their binge eating problem, and only one participant had gone through treatment for alcohol use. Commonly, the reason for not seeking care was not seeing alcohol as their major problem, not being asked about it in other healthcare settings or being too ashamed to talk about their problematic drinking.

*“Well*,* I’ve known for a long time that this is a problem. And it becomes double embarrassing in some way when you do*,* both things /… / and I think there are many who feel that you might have the courage and have the energy to seek help for one of them. But it’s like*,* it’s too much. Too sick. To have both things at the same time. But I have really thought several times that I would like to dare to talk about alcohol in particular.”* (Participant 11).

Among the ones who tried to bring up alcohol as a topic with their healthcare providers, the response was often, that they could not receive ED treatment if they had an alcohol problem. One participant explained how she was seeking care for her ED but was denied because of her problematic drinking.

*“They thought that I should get help with it (the alcohol) first. And then I was just disappointed and sad and then I ditched it all. Because it’s the same problem*,* I think. But it’s the binge eating that is my foundation*,* my comfort blanket that I’ve had for so very long. It felt like the completely wrong end to start”* (Participant 2).

One participant who were in treatment for her ED felt punished, as she was told to stop drinking to be able to continue receiving treatment.

*“My therapist told me that you have to stop drinking or you can’t continue here. Since I identify so strongly with my addictions*,* it felt like*,* rejecting that part of me meant rejecting me. You may not be able to face everything at once or break free from everything immediately*,* but that shouldn’t mean you get punished for it. Just because you haven’t stopped doing something yet doesn’t mean you should be denied treatment.”* (Participant 4).

Taken together, participants felt they did not receive adequate support in managing their problematic behaviors. They described feeling hesitant to bring up their alcohol use in healthcare settings. They also perceived that healthcare providers often failed to recognize alcohol use as relevant to their care and instead referred them to specialized addiction care, or other healthcare providers, regardless of what the patients considered their main problem.

## Discussion

The overall aim of this qualitative interview study was to investigate the experiences and perspectives of patients who struggle with both binge eating behaviors and problematic alcohol use. The study was done to more thoroughly understand how these problems are experienced, and whether they are perceived as interconnected and/ or maintained by each other. We concluded that binge eating, and problematic alcohol use were functionally connected through shared emotional antecedents. In addition, alcohol use contributed to maintaining the ED, as it was used to alleviate negative emotions both related to binge eating, but also creating short-term relief from ED-related cognitions, e.g. maintaining restriction, perfectionist attitudes towards oneself, thoughts of body shame, and physiological sensations of e.g. being full or hungry. Four themes were identified. Theme one described how binge eating and alcohol use served as emotion-regulation strategies. Theme two described the interplay between exerting control and experience of impaired control in both eating and drinking. The third theme addressed how the ED preceded the alcohol problem and how alcohol contributed to maintaining the ED. Finally, theme four captured how negative responses from healthcare providers on having a concurrent alcohol problem with the ED hampered adequate treatment seeking for both the ED and the alcohol problems.

All participants repeatedly described binge eating and alcohol use were preceded by strong emotions as antecedents, such as sadness, anhedonia, anxiety, stress, and irritability. More general descriptions were also used, such as general discomfort, and physical tension. These findings suggest that both binge eating and alcohol use are used as strategies to avoid and suppress negative affect. The later descriptions may indicate aspects of emotional unclarity, and difficulties identifying the actual affect. These findings are in line with previous research on the close relationship between binge eating as well as alcohol use being strategies for handling negative affect, and the role of negative reinforcement in maintaining both behaviors [13–15, 18, 41]. However, some participants also described how their eating and drinking episodes were triggered and sometimes maintained by positive affect, such as moments of celebration or as a form of self-reward. Whilst anticipating rewards or pursuing positive affect has been associated with alcohol use, the emphasis on alleviation of negative affect has been more prominent in explanations of binge eating [12, 15]. Ecological momentary assessment studies have demonstrated that negative emotions, and specifically guilt, increase just prior to a binge eating episode [15]. This study’s findings indicate that antecedents to binge eating may also be more neutral or positive feelings on some occasions.

The closely connected patterns of binge eating and alcohol consumption were characterized by the participants’ cyclic process between control and impaired control over both alcohol and eating, as described in theme two. Although participants reported having an ED before they had an alcohol problem, most reported longstanding difficulties limiting their alcohol intake, often drinking rapidly or with the occasional intention of losing control. Difficulties to limit, and drinking more than intended on a specific occasion, is a known predictor for developing more severe alcohol problems [42, 43]. Therefore, it is warranted to reach individuals with problem drinking, who may be at risk of developing more severe alcohol problems, and offer treatment in the context of their ongoing ED care. Addressing the alcohol problem when patients already sought treatment for their ED may reduce time to treatment, due to the stigma-related barrier of seeking treatment in the addiction services [22, 23].

Another complicating factor of the interaction of the ED and alcohol use was that loss of control over eating seemed to be more profound and resulted in longer and more intense binge episodes when drinking before or during the binge eating episode. The short-term effects of alcohol, which increase impulsivity, may therefore contribute to the severity of these situations and lead to even stronger post-binge eating reactions such as guilt, shame, and self-loathing. It may be hypothesized that such interactions between alcohol use and binge eating contribute to the heavier symptom burden in individuals with a comorbid ED and alcohol problems, as compared to those who have only an ED.

The third theme addressed how alcohol use came after the ED, and served as a maintainer of the ED. This is a novel finding with implications for prevention and treatment. Preventive efforts targeting binge eating may reduce the risk of engaging in problematic drinking among a subsample of individuals with eating disorders. As explained by the participants drinking was as a way of handling ED-related cognitions, and its related consequences. Typically, these situations were not related to binge eating. Instead, participants’ examples illustrated how alcohol functioned as avoidance of thoughts (e.g., self-critique and self-focus in social and intimate situations). Further, alcohol was used to create temporary “breaks” from intrusive thoughts about restricted eating. As these problem situations were not directly coupled with binge eating, it may be easier to miss from a clinician’s point of view. Awareness of drinking habits of patients and extending their daily monitoring of eating to alcohol use may provide functional insights into maintaining mechanisms perpetuating the ED and problematic alcohol use. Focus on triggers and functions of alcohol consumption may also help identifying cognitions and emotions that are related to eating, shape and weight, or self-worth that might otherwise not be as easily identified by the patient and the therapist. The perspective and experiences provided by the participants in this study also points to the importance of increasing focus on positive emotions in treatment and using stimulus control to ensure that positive emotions are experienced through activities that are incompatible with binge eating or dysfunctional alcohol use. The efficacy of focus on positive valence has been shown in other areas [44, 45]. In light of the findings from the current study, it is warranted to develop and evaluate the incremental effect of a module focusing on increasing positive valence in CBT-E. We suggest that a specific focus on positive valence is needed to go beyond the work on alternative activities incompatible with eating disorder behaviors, as outlined in the CBT-E manual [46].

In theme four, participants described how their attempts to bring up alcohol in their ED treatment were met with rejection, or a non-validating attitude from health care professionals. Being told that alcohol problems must be addressed first can result in reduced trust, feelings of being misunderstood, and not being seen as a whole person. These experiences resulted in patients either experiencing a disruption to the therapeutic alliance, or ending their ED treatment. From a clinician’s perspective, a lack of integrated methods, or uncertainty about whether it is part of their remit to address alcohol use, are identified barriers to systematically identifying and treating alcohol problems within psychiatric settings [47, 48]. It is not known if participants were identified by ED healthcare as having a severe or a more complex AUD, which demands specialized addiction treatment before the ED treatment. However, alcohol is a stigmatized condition, and being subjected to stereotypical views of alcohol problems, can reduce the willingness to seek specialized addiction care [22, 23]. It may be concluded that remitting patients with either hazardous use, or a mild to moderate AUD to specialized addiction care before being treated for their ED, can both prolong the time with ED, and the alcohol problem. This alco includes barriers to receiving inpatient-, or residential treatment for the ED, if the patient has an ongoing alcohol problem. Beyond outpatient settings, providers of residential care facilities may enhance treatment outcomes by integrating alcohol-related interventions into their programs. For medically stable patients, addressing both disorders concurrently in a residential setting might reduce barriers to care and prevent relapse in either condition.

### Strengths and limitations

There are strengths and limitations worth mentioning regarding the current study. To our knowledge, this is the first qualitative study with the specific aim to explore the functional relationship between binge eating and alcohol problems from patients’ perspective, for the purpose of developing a novel treatment aiming to treat both conditions in an integrated approach. The study contributed important information from patients’ perspectives on the development, relationship between, and the maintenance of EDs and comorbid alcohol problems. The research team involves clinicians with longstanding experience from both the ED and AUD fields. Previous subjective experiences may have influenced the interpretation of the data. To balance the impact of such subjectivity, three researchers were involved in coding the material, and the rest of the team contributed by supporting the process of developing themes that accurately represent the data. Another limitation is the uneven gender distribution, which may have influenced the results, given that men more commonly have alcohol problems. Many ED treatment services many may not be gender inclusive- or affirmative, which may be a treatment barrier, to those not identifying as women, and such lack of gender inclusivity may have shaped the sample in the study. The gender distribution of participants did mirror the typical distribution of a higher percentage of women men vs women seeking treatment for their ED, as compared to men and non-binary individuals.

## Conclusions

The findings demonstrate that co-occurring binge eating and problematic alcohol use can be expected to be closely interconnected. In our study, participants reported that alcohol problems developed after the eating disorder and contributed to its maintenance, both as an emotion-regulation strategy, as part of the binge eating cycle, and by providing short-term relief from ED-related cognitions. These data support previous suggestions of the need for integrated treatment approaches that address both behaviors simultaneously [49–52]. Having a history of AUD may be a barrier to receiving accurate psychiatric treatment including eating disorders [53]. International guidelines recommend concurrent treatment in individuals with a psychiatric disorder, such as bulimia nervosa, in combination with mild to moderate alcohol problems [54]. This demands further education and availability of integrated methods for comorbid conditions [55]. For individuals with more complex alcohol problems, patients typically need treatment for their alcohol problem first. Thereafter, to overcome stigma-related barriers to ED services, they may need specific support in their referral to ED care, such as a letter of advocacy.

Our results suggest that it is motivated to work, not only simultaneously, but with an integrated treatment that specifically addresses the possibly similar and connected maintenance processes of the conditions. Similar work has been done in the field, for eating disorders and other complex conditions, such as post-traumatic stress disorder, which can be of guidance for further methodological development [56]. Future qualitative research on treatment seeking individuals recruited from addiction services, who also present with eating disorder symptoms, would further improve our understanding of the functional relationship between eating- and alcohol use disorders. Further, it is needed to focus on preventive efforts through earlier identification of alcohol problems in patients with binge eating problems.

## Supplementary Information

Below is the link to the electronic supplementary material.

Supplementary Material 1.

## Data Availability

Data can be available upon reasonable request.

## References

[1] Galmiche M, Déchelotte P, Lambert G, Tavolacci MP. Prevalence of eating disorders over the 2000–2018 period: a systematic literature review. Am J Clin Nutr. 2019;109(5):1402–13. 31051507 10.1093/ajcn/nqy342

[2] Silén Y, Keski-Rahkonen A. Worldwide prevalence of DSM-5 eating disorders among young people. Curr Opin Psychiatry [Internet]. 2022;35(6). Available from: https://journals.lww.com/co-psychiatry/fulltext/2022/11000/worldwide_prevalence_of_dsm_5_eating_disorders.3.aspx 10.1097/YCO.0000000000000818 36125216

[3] Mellentin AI, Skøt L, Guala MM, Støving RK, Ascone L, Stenager E, et al. Does receiving an eating disorder diagnosis increase the risk of a subsequent alcohol use disorder? A Danish nationwide register-based cohort study. Addict Abingdon Engl. 2022;117(2):354–67. 10.1111/add.15639 34251067

[4] Treasure J, Duarte TA, Schmidt U. Eating disorders. Lancet Lond Engl. 2020;395(10227):899–911. 10.1016/S0140-6736(20)30059-3 32171414

[5] Bulik CM, Klump KL, Thornton L, Kaplan AS, Devlin B, Fichter MM, et al. Alcohol use disorder comorbidity in eating disorders: a multicenter study. J Clin Psychiatry. 2004;65(7):1000–6. 15291691 10.4088/jcp.v65n0718

[6] Forsén Mantilla E, Clinton D, Monell E, Levallius J, Birgegård A. Impulsivity and compulsivity as parallel mediators of emotion dysregulation in eating-related addictive-like behaviors, alcohol use, and compulsive exercise. Brain Behav. 2022;12(1):e2458. 34928542 10.1002/brb3.2458 PMC8785615

[7] Hirvelä L, Sipilä PN, Keski-Rahkonen A. Relationship between sensation seeking, alcohol problems and bulimic symptoms: a community-based, longitudinal study. Eat Weight Disord - Stud Anorex Bulim Obes. 2022;27(2):589–95. 10.1007/s40519-021-01193-6 PMC8933307 33900563

[8] Munn-Chernoff MA, Editorial. Why Assessing Co-Occurring Eating Disorders and Alcohol Use in Sexual Minority Youth Is Important. J Am Acad Child Adolesc Psychiatry. 2019;58(2):154–5. 30738540 10.1016/j.jaac.2018.11.006

[9] Munn-Chernoff MA, Johnson EC, Chou YL, Coleman JRI, Thornton LM, Walters RK, et al. Shared genetic risk between eating disorder- and substance-use-related phenotypes: Evidence from genome-wide association studies. Addict Biol. 2021;26(1):e12880. 32064741 10.1111/adb.12880 PMC7429266

[10] Baker JH, Thornton LM, Strober M, Brandt H, Crawford S, Fichter MM, et al. Temporal sequence of comorbid alcohol use disorder and anorexia nervosa. Addict Behav. 2013;38(3):1704–9. 23254222 10.1016/j.addbeh.2012.10.005 PMC3558554

[11] Franko DL, Dorer DJ, Keel PK, Jackson S, Manzo MP, Herzog DB. How do eating disorders and alcohol use disorder influence each other? Int J Eat Disord. 2005;38(3):200–7. 16216020 10.1002/eat.20178

[12] Borm IM, Hartmann S, Barnow S, Pruessner L. Binge eating as emotion regulation? A meta-analysis of ecological momentary assessment studies. Clin Psychol Rev. 2025;121:102625. 40768884 10.1016/j.cpr.2025.102625

[13] Gorka SM, Hedeker D, Piasecki TM, Mermelstein R. Impact of alcohol use motives and internalizing symptoms on mood changes in response to drinking: An ecological momentary assessment investigation. Drug Alcohol Depend. 2017;173:31–8. 28189925 10.1016/j.drugalcdep.2016.12.012 PMC5366258

[14] Pisetsky EM, Crosby RD, Cao L, Fitzsimmons-Craft EE, Mitchell JE, Engel SG, et al. An examination of affect prior to and following episodes of getting drunk in women with bulimia nervosa. Psychiatry Res. 2016;240:202–8. 27111214 10.1016/j.psychres.2016.04.044 PMC4939267

[15] Schaefer LM, Smith KE, Anderson LM, Cao L, Crosby RD, Engel SG, et al. The role of affect in the maintenance of binge-eating disorder: Evidence from an ecological momentary assessment study. J Abnorm Psychol. 2020;129(4):387–96. 32212743 10.1037/abn0000517 PMC7174093

[16] Fischer S, Settles R, Collins B, Gunn R, Smith GT. The role of negative urgency and expectancies in problem drinking and disordered eating: testing a model of comorbidity in pathological and at-risk samples. Psychol Addict Behav J Soc Psychol Addict Behav. 2012;26(1):112–23. 10.1037/a0023460 PMC3954822 21604832

[17] Jakubczyk A, Trucco EM, Kopera M, Kobyliński P, Suszek H, Fudalej S, et al. The association between impulsivity, emotion regulation, and symptoms of alcohol use disorder. J Subst Abuse Treat. 2018;91:49–56. 29910014 10.1016/j.jsat.2018.05.004 PMC6020846

[18] Prefit AB, Cândea DM, Szentagotai-Tătar A. Emotion regulation across eating pathology: A meta-analysis. Appetite. 2019;143:104438. 31479694 10.1016/j.appet.2019.104438

[19] Escrivá-Martínez T, Herrero R, Molinari G, Rodríguez-Arias M, Verdejo-García A, Baños RM. Binge Eating and Binge Drinking: A Two-Way Road? An Integrative Review. Curr Pharm Des. 2020;26(20):2402–15. 32175840 10.2174/1381612826666200316153317

[20] Cobbaert L, Millichamp AR, Elwyn R, Silverstein S, Schweizer K, Thomas E, et al. Neurodivergence, intersectionality, and eating disorders: a lived experience-led narrative review. J Eat Disord. 2024;12(1):187. 39568093 10.1186/s40337-024-01126-5 PMC11580580

[21] Wiśniewski P, Jakubczyk A, Trucco EM, Kobyliński P, Suszek H, Zaorska J, et al. Interoception, alexithymia, and anxiety among individuals with alcohol use disorder. Front Psychiatry. 2023;14:1229985. 37810600 10.3389/fpsyt.2023.1229985 PMC10556496

[22] Wallhed Finn S, Bakshi AS, Andréasson S. Alcohol consumption, dependence, and treatment barriers: perceptions among nontreatment seekers with alcohol dependence. Subst Use Misuse. 2014;49(6):762–9. 24601784 10.3109/10826084.2014.891616

[23] Probst C, Manthey J, Martinez A, Rehm J. Alcohol use disorder severity and reported reasons not to seek treatment: a cross-sectional study in European primary care practices. Subst Abuse Treat Prev Policy. 2015;10:32. 26264215 10.1186/s13011-015-0028-z PMC4534056

[24] Ali K, Farrer L, Fassnacht DB, Gulliver A, Bauer S, Griffiths KM. Perceived barriers and facilitators towards help-seeking for eating disorders: A systematic review. Int J Eat Disord. 2017;50(1):9–21. 27526643 10.1002/eat.22598

[25] Nicula M, Pellegrini D, Grennan L, Bhatnagar N, McVey G, Couturier J. Help-seeking attitudes and behaviours among youth with eating disorders: a scoping review. J Eat Disord. 2022;10(1):21. 35164872 10.1186/s40337-022-00543-8 PMC8845232

[26] Hepworth N, Paxton SJ. Pathways to help-seeking in bulimia nervosa and binge eating problems: A concept mapping approach. Int J Eat Disord. 2007;40(6):493–504. 17573682 10.1002/eat.20402

[27] Dansky BS, Brewerton TD, Kilpatrick DG. Comorbidity of bulimia nervosa and alcohol use disorders: Results from the National Women’s Study. Int J Eat Disord. 2000;27(2):180–90. 10657891 10.1002/(sici)1098-108x(200003)27:2<180::aid-eat6>3.0.co;2-z

[28] Tong A, Sainsbury P, Craig J. Consolidated criteria for reporting qualitative research (COREQ): a 32-item checklist for interviews and focus groups. Int J Qual Health Care. 2007;19(6):349–57. 17872937 10.1093/intqhc/mzm042

[29] Sobell LC, Sobell MB. Timeline follow-back: A technique for assessing self-reported alcohol consumption. Measuring alcohol consumption: Psychosocial and biochemical methods. Springer; 1992. pp. 41–72.

[30] Sysko R, Walsh BT, Fairburn CG. Eating Disorder Examination-Questionnaire as a measure of change in patients with bulimia nervosa. Int J Eat Disord. 2005;37(2):100–6. 15732070 10.1002/eat.20078

[31] Bohn K, Doll HA, Cooper Z, O’Connor M, Palmer RL, Fairburn CG. The measurement of impairment due to eating disorder psychopathology. Behav Res Ther. 2008;46(10):1105–10. 18710699 10.1016/j.brat.2008.06.012 PMC2764385

[32] Braun V, Clarke V. Using thematic analysis in psychology. Qual Res Psychol. 2006;3(2):77–101.

[33] Braun V. One size fits all? What counts as quality practice in (reflexive) thematic analysis? Qual Res Psychol. 2021;18(3):328–52.

[34] Saunders JJB, AASLAND OG, BABOR TF, DE LA FUENTE JR GRANTM. Development of the Alcohol Use Disorders Identification Test (AUDIT): WHO Collaborative Project on Early Detection of Persons with Harmful Alcohol Consumption-II. Addict Abingdon Engl. 1993;88(6):791–804. 10.1111/j.1360-0443.1993.tb02093.x 8329970

[35] Bjureberg J, Ljótsson B, Tull MT, Hedman E, Sahlin H, Lundh LG, et al. Development and Validation of a Brief Version of the Difficulties in Emotion Regulation Scale: The DERS-16. J Psychopathol Behav Assess. 2016;38(2):284–96. 27239096 10.1007/s10862-015-9514-x PMC4882111

[36] Spitzer RL, Kroenke K, Williams JB. Validation and utility of a self-report version of PRIME-MD: the PHQ primary care study. Primary Care Evaluation of Mental Disorders. Patient Health Questionnaire JAMA. 1999;282(18):1737–44. 10568646 10.1001/jama.282.18.1737

[37] Spitzer RL, Kroenke K, Williams JBW, Löwe B. A brief measure for assessing generalized anxiety disorder: the GAD-7. Arch Intern Med. 2006;166(10):1092–7. 16717171 10.1001/archinte.166.10.1092

[38] Braun V, Clarke V. Thematic analysis: A practical guide. 2021.

[39] SPSS Statistics. IBM Corp., developer. IBM SPSS Statistics for Windows [computer software].Version 31.0. Armonk, New York: IBM Corp. 2017. Available: https://www.ibm.com/products/spss-statistics

[40] Malterud K, Siersma VD, Guassora AD. Sample size in qualitative interview studies: guided by information power. Qual Health Res. 2016;26(13):1753–60. 26613970 10.1177/1049732315617444

[41] Berg KC, Crosby RD, Cao L, Peterson CB, Engel SG, Mitchell JE, et al. Facets of negative affect prior to and following binge-only, purge-only, and binge/purge events in women with bulimia nervosa. J Abnorm Psychol. 2013;122(1):111–8. 22985015 10.1037/a0029703 PMC3646562

[42] Tuithof M, ten Have M, van den Brink W, Vollebergh W, de Graaf R. Alcohol consumption and symptoms as predictors for relapse of DSM-5 alcohol use disorder. Drug Alcohol Depend. 2014;140:85–91. 24793368 10.1016/j.drugalcdep.2014.03.035

[43] Gruenewald PJ, Caetano R, Mair C. Impacts of impaired control and pharmacological criteria for lifetime alcohol use disorder on relationships between drinking and problems. Drug Alcohol Depend. 2025;276:112910. 41076969 10.1016/j.drugalcdep.2025.112910 PMC12541909

[44] Craske MG, Dunn BD, Meuret AE, Rizvi SJ, Taylor CT. Positive affect and reward processing in the treatment of depression, anxiety and trauma. Nat Rev Psychol. 2024;3(10):665–85.

[45] Sandman CF, Craske MG. Psychological Treatments for Anhedonia. Curr Top Behav Neurosci. 2022;58:491–513. 34935116 10.1007/7854_2021_291

[46] Fairburn, Christopher R, Straebler S, Cooper Z, Murphy R. Cognitive behavioral therapy for eating disorders. Psychiatr Clin. 2010;33(3):611–27. 10.1016/j.psc.2010.04.004 PMC2928448 20599136

[47] Sundbye J, Hammarberg A, Guterstam J, Salomonsson S, Finn SW. A qualitative study of patient’s experiences of receiving alcohol interventions according to the 15-method in psychiatric care. BMC Psychiatry. 2025;25(1):1031. 41152812 10.1186/s12888-025-07553-1 PMC12570522

[48] Evans J, Horn K, Cowan D, Brunero S. Development of a clinical pathway for screening and integrated care of eating disorders in a rural substance use treatment setting. Int J Ment Health Nurs. 2020;29(5):878–87. 32233124 10.1111/inm.12722

[49] Gregorowski C, Seedat S, Jordaan GP. A clinical approach to the assessment and management of co-morbid eating disorders and substance use disorders. BMC Psychiatry. 2013;13:289. 24200300 10.1186/1471-244X-13-289 PMC4226257

[50] Sysko R, Hildebrandt T. Cognitive-behavioural therapy for individuals with bulimia nervosa and a co-occurring substance use disorder. Eur Eat Disord Rev. 2009;17(2):89–100. 19130465 10.1002/erv.906 PMC2990777

[51] Harrop EN, Marlatt GA. The comorbidity of substance use disorders and eating disorders in women: Prevalence, etiology, and treatment. Addict Behav. 2010;35(5):392–8. 20074863 10.1016/j.addbeh.2009.12.016

[52] Brewerton TD, Dennis AB. Eating disorders, addictions and substance use disorders: Research, clinical and treatment perspectives. Springer; 2014.

[53] Petersén E, Thurang A, Berman AH. Staff experiences of encountering and treating outpatients with substance use disorder in the psychiatric context: a qualitative study. Addict Sci Clin Pract. 2021;16(1):29–11. 33971959 10.1186/s13722-021-00235-9 PMC8112046

[54] Torrens M, Mestre-Pintó JI, Domingo-Salvany A. European Monitoring Centre for Drugs and Drug Addiction issuing body. Comorbidity of substance use and mental disorders in Europe. Luxembourg: Publication Office of the European Union; 2015. (Insights, 19).

[55] Kools N, Rozema AD, van den Bulck FAE, Bovens RHLM, Mathijssen JJP, van de Mheen D. Exploring barriers and facilitators to addressing hazardous alcohol use and AUD in mental health services: a qualitative study among Dutch professionals. Addict Sci Clin Pract. 2024;19(1):65. 39252050 10.1186/s13722-024-00497-z PMC11385808

[56] Trottier K, Monson CM, Wonderlich SA, Crosby RD. Results of the first randomized controlled trial of integrated cognitive-behavioral therapy for eating disorders and posttraumatic stress disorder. Psychol Med. 2022;52(3):587–96. 34872625 10.1017/S0033291721004967 PMC8883823

