# Supplementary material for: I am in the passenger seat of my own body: a qualitative interview study of the relationship between binge eating and concurrent problematic alcohol use
Source: J Eat Disord. 2026 Mar 16;14:83. doi: 10.1186/s40337-026-01576-z (PMC13064080; doi:10.1186/s40337-026-01576-z)
Supplement: Supplementary file 1 — Supplementary Material 1. [file 40337_2026_1576_MOESM1_ESM.docx]

SUPPLEMENTARY FILE 1.

Interview guide

Tell me about your eating disorder and your perceived alcohol problems

How do you perceive your binge eating and alcohol problem?

Do you find your problems to be connected in any way?

If so, how?

Are the problems maintaining each other? If so, how?
